# Evaluating Cardiovascular Risks: The Platelet Lymphocyte Ratio and the Neutrophil Lymphocyte Ratio As High-Risk Heart Score Predictors in Non-ST Elevation Myocardial Infarction (NSTEMI) and Unstable Angina Patients

**DOI:** 10.7759/cureus.61279

**Published:** 2024-05-28

**Authors:** Jibin Simon, Srinivasa Guptha, Kandasamy Venkataraju Rajalakshmi, Ananthakumar Perumal Kumaresan, Sharan Bose

**Affiliations:** 1 General Medicine, Saveetha Medical College and Hospitals, Saveetha Institute of Medical and Technical Sciences, Saveetha University, Chennai, IND

**Keywords:** heart score, neutrophil lymphocyte ratio, platelet lymphocyte ratio, acute coronary syndrome, non-st elevation myocardial infarction, unstable angina

## Abstract

Introduction

Acute coronary syndromes (ACS), encompassing non-ST elevation myocardial infarction (NSTEMI) and unstable angina (UA), present significant challenges in risk assessment and management, particularly in resource-constrained environments like India. The burden of cardiovascular diseases in such regions necessitates cost-effective and readily accessible tools for risk stratification. Previous research has emphasized the role of inflammatory markers in coronary artery disease (CAD), prompting investigations into simple and affordable biomarkers for risk assessment. Platelet lymphocyte ratio (PLR) and neutrophil lymphocyte ratio (NLR) have emerged as potential biomarkers for thrombotic activity in cardiac illnesses, offering simplicity, accessibility, and cost-effectiveness in risk assessment making them particularly valuable in resource-poor settings like India, where advanced diagnostic tools may be limited.

Objective

This study aims to evaluate the effectiveness of PLR and NLR as predictors of high-risk HEART (history, ECG, age, risk factors, and troponin) scores in patients with NSTEMI and UA.

Methods

A prospective cross-sectional study was conducted at the Saveetha Medical College and Hospitals in Chennai, India, from March 2021 to September 2022. The study included 288 adults diagnosed with NSTEMI or UA, aged 18 years and above. The inclusion criteria comprised patients with confirmed diagnoses of NSTEMI or UA based on clinical symptoms, electrocardiographic findings, and cardiac biomarker elevation. The exclusion criteria encompassed patients with active infections, acute traumatic injuries, end-stage renal disease, malignant neoplasms, and ST-elevation myocardial infarction (STEMI). In addition to the HEART score, PLR, and NLR were computed to assess the prognosis of patients admitted to the Saveetha Medical College and Hospitals.

Results

The statistical analysis revealed significant correlations between PLR, NLR, and HEART score risk categories. The Pearson's correlation coefficient indicated strong associations between PLR/NLR values and HEART score risk groups, suggesting their potential as predictive markers for adverse clinical outcomes. Additionally, analysis of variance (ANOVA) demonstrated significant differences in PLR/NLR values across different HEART score risk categories, further highlighting their relevance in risk stratification. The effect sizes for these correlations were moderate to large, indicating clinically meaningful associations between PLR/NLR and cardiovascular risk.

Conclusion

In cases of NSTEMI and UA, PLR and NLR show potential as simple and inexpensive indicators of high-risk patients. By leveraging these inexpensive biomarkers, healthcare providers can enhance risk assessment and prognostication in patients presenting with ACS, facilitating timely interventions and tailored management strategies.

## Introduction

One of the main causes of death and disability worldwide is acute coronary syndrome (ACS), and patients with multi-vessel coronary artery disease (CAD) and co-morbidities are more likely to die than those without ST-segment elevation [1]. In non-ST elevation myocardial infarction (NSTEMI), CAD diagnosis and prediction might be extremely challenging [2]. There has previously been a great deal of research done on the connection between inflammatory indicators and CAD [3]. One key mechanism for the rupture of weak plaques is inflammation. Numerous inflammatory cells, primarily neutrophils, proliferate within the coronary artery atheromatous plaque and concentrate within the channel wall. This process induces vascular endothelial cells to adhere, become activated, and secrete factors that damage tissue, including alkaline phosphatase and peroxidase [4].

In cardiac diseases, the platelet-to-lymphocyte ratio (PLR) and neutrophil-to-lymphocyte ratio (NLR) were presented as potential biomarkers for increased thrombotic activity [5]. It has been demonstrated that NLR and PLR are reasonably simple, rapid, and affordable methods for assessing the risk of ACS in patients as well as aiding in diagnosis [6]. Previously, Wikananda et al. [7] investigated the link between the NLR ratio and the Global Registry of Acute Coronary Events (GRACE) score in patients with acute myocardial infarction. They discovered that the NLR was considerably higher in the high-risk group. Similarly, Acet et al. [8] investigated the link between NLR and thrombolysis in myocardial infarction (TIMI) risk score in a sample of ST-elevation myocardial infarction (STEMI) patients and discovered that NLR was positively correlated with TIMI risk score. The true predictive performance of the HEART (history, ECG, age, risk factors, and troponin) score was investigated in a retrospective multicenter study published in 2010, which summarized the findings from numerous external validation studies. A HEART score over the low-risk threshold (≥4) showed strong sensitivity (95.9%) for short-term major adverse cardiovascular events (MACEs), short-term mortality (95.0%), and myocardial infarction (97.5%), whereas a high-risk HEART score (≥7) showed high specificity (95.0%) for short-term MACE. This study supports the use of the HEART for risk classification of individuals who present with chest discomfort [9]. Research on using the NLR and PLR ratio as a high-risk HEART score predictor in NSTEMI and/or unstable angina (UA) is insufficient. While previous studies have explored the association between inflammatory markers and CAD, there is limited research focusing on the predictive utility of PLR and NLR in the context of high-risk HEART score patients.

The rationale for selecting PLR and NLR lies in their simplicity, rapidity, and affordability as biomarkers, making them particularly suitable for risk assessment, especially in resource-constrained settings where comprehensive scoring systems like HEART and TIMI may not be feasible due to the unavailability of investigations like troponin. Unlike these traditional scoring systems, which rely on multiple parameters including troponin levels that necessitate specialized equipment and expertise, PLR and NLR can be easily calculated from routine complete blood count (CBC) data. 

By integrating previous research findings, such as the association between inflammatory markers and CAD, this study aims to contribute to a comprehensive understanding of the pathophysiological mechanisms underlying ACS. Furthermore, by elucidating the role of PLR and NLR as predictive markers in high-risk HEART score patients, the study seeks to provide clinicians with valuable tools for early risk assessment and intervention in NSTEMI and UA cases.

## Materials and methods

The study was conducted in the Department of General Medicine at the Saveetha Medical College and Hospitals, Chennai, India, from March 2021 to September 2022. The duration of the study was 18 months. This study utilized a prospective cross-sectional design to evaluate the prognosis of patients admitted to the Saveetha Medical College and Hospitals with NSTEMI or UA. The cross-sectional design enabled the assessment of data at a specific moment in time and facilitated the simultaneous evaluation of multiple variables, thereby allowing for a comprehensive analysis of prognostic factors in ACS. The approval for the study was obtained from the Saveetha Medical College and Hospitals Institutional Ethics Committee (SMC/IEC/2021/03/001).

A total of 288 individuals aged 18 years and above, diagnosed with NSTEMI or UA, were recruited from the General Medicine Department of Saveetha Medical College and Hospitals. The inclusion criteria encompassed patients with confirmed diagnoses of NSTEMI or UA based on clinical symptoms, electrocardiographic (ECG) findings, and cardiac biomarker elevation. The diagnostic criteria for NSTEMI and UA primarily involve a combination of clinical symptoms, ECG findings, and cardiac biomarker levels. NSTEMI is characterized by symptoms such as chest pain or discomfort, ECG changes like ST-segment depression, and elevated cardiac biomarkers indicating myocardial injury. On the other hand, UA presents with similar symptoms but lacks evidence of myocardial injury on cardiac biomarker testing, distinguishing it from NSTEMI.

In the context of a study investigating the prognostic value of PLR and NLR in patients with ACS, the following patients were excluded to minimize the confounding factors that could potentially influence the ratios and bias the results: patients with active infections were excluded from the study due to the known effect of infection on peripheral blood cell counts, including neutrophils and lymphocytes. Excluding patients with active infections ensured that the measured biomarker ratios were not confounded by transient alterations related to an acute inflammatory response. Patients with trauma, particularly those with acute traumatic injuries such as fractures and burns or those undergoing major surgeries, can induce a systemic inflammatory response characterized by leukocytosis and alterations in platelet counts. Therefore, such patients were excluded from the study. Patients with end-stage renal disease were excluded as they often exhibit alterations in leukocyte and platelet counts due to chronic inflammation, uremia, and comorbidities such as hypertension and diabetes. Patients with malignant neoplasms were excluded as they are associated with various systemic effects, including inflammation, immune dysregulation, and hematopoietic alterations. Patients with STEMI were also excluded to focus specifically on patients with NSTEMI or UA. STEMI and NSTEMI/UA represent distinct subsets of ACS with different pathophysiological mechanisms and prognostic implications. Excluding patients with STEMI allowed for a more homogeneous study population and facilitated a focused evaluation of PLR and NLR in the context of NSTEMI and UA.

A sample size of 288 patients was selected to ensure sufficient statistical power for the analysis, allowing for robust conclusions to be drawn from the data. A non-probability convenience sampling approach was utilized, where patients were selected based on their availability and willingness to participate. While this method may introduce some degree of selection bias, it was practical within the constraints of the study setting and facilitated efficient data collection. Given the urgent nature of ACS, timely recruitment of participants was essential for capturing real-world data.

Data collection procedures were standardized and implemented consistently across all participants to ensure data reliability and validity. Patients meeting the inclusion criteria were identified upon admission to the Saveetha Medical College and Hospitals. The recruitment was conducted in accordance with the study protocol and ethical guidelines. Prior to any data collection procedures, the study was explained to each participant in their local language, and informed consent was obtained from each participant or their legally authorized representative. Detailed demographic information, including age, gender, and relevant medical history (e.g., hypertension, diabetes, dyslipidemia), was collected from each participant through interviews and review of medical records. Upon admission, patients underwent thorough clinical evaluations, including physical examinations, vital sign measurements, and ECG assessments. Clinical symptoms, such as chest pain characteristics and associated features, were documented to aid in risk stratification and diagnosis. 

Blood samples were collected from each participant following standardized procedures to ensure consistency and accuracy. Venipuncture was performed by trained phlebotomists using aseptic techniques to minimize contamination. The samples were then processed promptly to prevent cellular degradation and analyzed using automated hematology analyzers calibrated according to manufacturer specifications. The calibration procedures were conducted regularly to maintain the accuracy and precision of hematological measurements. CBC was done to determine absolute neutrophil, lymphocyte, and platelet counts. These parameters were used to compute the PLR and NLR, key prognostic indicators in the study. To ensure the reliability and validity of laboratory data, rigorous quality control measures were implemented throughout the study. This included daily calibration of laboratory instruments, verification of analyzer performance using commercial quality control materials, and adherence to established standard operating procedures for sample handling and analysis. Additionally, trained laboratory personnel regularly assessed instrument accuracy and precision, promptly addressing any deviations to minimize potential sources of error.

The HEART score, comprising history, electrocardiogram, age, risk factors, and troponin levels, was calculated for each participant based on clinical data and test results. All collected data, including clinical assessments, laboratory results, and HEART score calculations, were meticulously documented and entered into a secure database. Measures were taken to ensure data accuracy, completeness, and confidentiality throughout the study period. The statistical analysis in this study utilized IBM SPSS Statistics for Windows, Version 20 (Released 2011; IBM Corp., Armonk, New York, United States). The Student's t-test was utilized to compare means of continuous variables, such as age or laboratory values, between groups, while the Chi-square test assessed associations between categorical variables, like gender or comorbidities, and clinical outcomes. The frequency distribution summarized the occurrence of categorical or ordinal variables, such as HEART score categories or risk factor prevalence. Pearson's correlation coefficient gauged the linear relationship between continuous variables, examining connections between PLR/NLR and clinical outcomes. ANOVA (analysis of variance) formula was employed for multivariate analysis, assessing differences among multiple independent variables, such as PLR, NLR, and HEART score components, in relation to a dependent variable, such as major adverse cardiac events. No adjustments were made for multiple comparisons or controlling for potential confounding variables in this analysis.

To maintain data integrity, quality control measures were implemented at each stage of the data collection process. Quality control measures were rigorously implemented throughout the study to ensure data accuracy, completeness, and confidentiality. Training sessions were conducted for study personnel to familiarize them with the study protocol, data collection procedures, and ethical guidelines. These sessions emphasized the importance of adhering to standardized protocols and maintaining participant confidentiality. Data collection protocols were standardized to ensure consistency across all study participants. Detailed instructions were provided to study personnel regarding the conduct of clinical evaluations, laboratory analyses, and HEART score calculations. Regular monitoring and supervision were carried out to ensure adherence to these protocols and to address any potential issues or concerns. Audit procedures were implemented to monitor the integrity of the data collection process. Periodic audits were conducted to identify and rectify any discrepancies or errors in data entry or documentation. These audits served to validate the accuracy and completeness of the collected data and enhance the overall reliability of the study findings. To maintain participant confidentiality and adhere to ethical guidelines, strict measures were implemented. All study personnel were required to sign confidentiality agreements to protect participant privacy. Data were stored securely in a password-protected database accessible only to authorized personnel. Identifiable information was anonymized to ensure participant confidentiality throughout the study period. Overall, these quality control measures demonstrated the study's commitment to maintaining data integrity and adhering to ethical principles. By implementing rigorous training, standardized protocols, audit procedures, and confidentiality measures, the study ensured the reliability and validity of the collected data while safeguarding participant confidentiality.

## Results

The study investigated the link between HEART score risk categories and hematological parameters in patients with NSTEMI and UA. Based on their HEART scores, 288 patients were divided into three risk categories: low, moderate, and high. While there was no significant link between clinical syndrome and the HEART score risk category, hematological investigations revealed some connections. Elevated neutrophil count and NLR were found in higher-risk groups, indicating an inflammatory component in cardiac risk assessment. Lower lymphocyte count and PLR were related to higher-risk categories, demonstrating a role for immunological dysfunction and platelet activity in predicting negative outcomes.

Table 1 presents a breakdown of the HEART score risk categories found in the study population. According to the data, the majority of patients (42.7%) were classified as low-risk. In contrast, a smaller proportion of patients were classified as moderate (32.6%) or high-risk (24.7%). Our study had a high proportion of low-risk patients compared to other risk categories. These findings underscore the variability of risk profiles within the research group, with a significant subset of patients presenting with higher-risk traits, which may merit closer monitoring and more aggressive management options.

**Table 1:** Frequency distribution of HEART score risk category in the study HEART: history, ECG, age, risk factors, and troponin Low risk: HEART score 0-3; moderate risk: HEART score 4-6; high risk: HEART score more than or equal to 7

HEART score risk category	Frequency	Percentage
Low risk	123	42.7
Moderate risk	94	32.6
High risk	71	24.7
Total	288	100

Table 2 presents the number and proportion of patients in each group who had NSTEMI or UA. The data reveal a consistent pattern across all three groups, with NSTEMI being the most common clinical condition observed. Group 1 has the largest proportion of NSTEMI cases (73.9%), followed closely by Groups 2 and 3 (73.4% and 73.2%, respectively). In contrast, the incidence of UA is lower in all groups, ranging from 26.1% to 26.8%. Overall, the distribution of clinical syndromes does not appear to change significantly within HEART score risk categories, as demonstrated by the non-significant Pearson's Chi-square test result (χ² = 0.16, p = 0.992). This suggests the likelihood of presenting with NSTEMI or UA does not differ significantly based on the initial risk assessment using the HEART score. Such findings have important implications for clinical practice. While the HEART score is widely used for risk stratification in patients with suspected ACS, our results suggest that relying solely on this scoring system may not accurately predict the specific clinical syndrome a patient will present with.

**Table 2:** Comparison of clinical syndrome and HEART score risk category by using Chi-square test Group 1: low-risk HEART score patients, Group 2: moderate-risk HEART score patients, Group 3: high-risk HEART score patients; NSTEMI: non-ST elevation myocardial infarction; HEART: history, ECG, age, risk factors, and troponin Pearson’s Chi-square test: χ² = 0.16, p = 0.992 (ns), while 'ns' indicates that the result is not statistically significant and the sample size is represented as 'N'

Clinical syndrome	Group 1 N (%)	Group 2 N (%)	Group 3 N (%)	Total N (%)
NSTEMI	91 (73.9)	69 (73.4)	52 (73.2)	212 (73.6)
Unstable angina	32 (26.1)	25 (26.6)	19 (26.8)	76 (26.4)
Total	123 (100)	94 (100)	71 (100)	288 (100)

Table 3 represents the mean and standard deviation (SD) of white blood cell (WBC) counts for three groups based on the HEART score risk category. Group 1 includes individuals designated as low-risk, Group 2 as moderate-risk, and Group 3 as high-risk. The sample sizes (N) for each category are also specified. As the HEART score risk group increases, the mean WBC count decreases. Group 1 has the highest mean count of 12.514 x 10^9/L, followed by Group 2 with a mean of 11.620 x 10^9/L, and Group 3 with the lowest mean of 11.09 x 10^9/L. These mean differences are supported by the estimated F-value of 4.283, which indicates that WBC counts vary between groups. Furthermore, the p-value associated with the ANOVA is 0.015, indicating a statistically significant difference in WBC counts between the three groups. This implies that the WBC count may vary greatly depending on the initial risk assessment using the HEART score, with lower numbers indicating greater risk categories. The findings suggest a possible link between WBC count and the HEART score risk category, with lower WBC counts reported in patients classified as higher risk. Lower WBC counts in higher-risk groups may indicate a dysregulated inflammatory response in these patients. Furthermore, lower WBC counts in higher-risk groups may also be indicative of bone marrow suppression or immunosuppression, which are known to occur in patients with severe cardiovascular disease. These mechanisms could lead to decreased production or increased consumption of WBCs, resulting in lower circulating counts.

**Table 3:** Comparison of white blood corpuscle and HEART score risk category by using one-way ANOVA Group 1: low-risk HEART score patients, Group 2: moderate-risk HEART score patients, Group 3: high-risk HEART score patients; 95% CI: confidence intervals (95%); HEART: history, ECG, age, risk factors, and troponin; ANOVA: analysis of variance Values are expressed in mean ± SD and the sample size is represented as 'N' One-way ANOVA test: *p < 0.05, **p < 0.001 (statistically significant), ns (not significant)

Parameter	Group 1 - Mean ± SD (95% CI), N = 123	Group 2 - Mean ± SD (95% CI), N = 94	Group 3 - Mean ± SD (95% CI), N = 71	F-value	p-value
White blood corpuscle (10^9^/L)	12.514 ± 3.46 (11.864 - 13.164)	11.620 ± 3.818 (10.837 - 12.403)	11.09 ± 2.837 (10.269 - 11.911)	4.283	0.015*

Table 4 represents the mean and SD of neutrophil counts for three groups categorized by the HEART score risk category. The mean neutrophil count rises with each HEART score risk category, with Group 1 having the lowest mean count of 70.80%, followed by Group 2 with a mean of 77.59%, and Group 3 with the highest mean of 82.36%. The estimated F-value of 46.350 supports these mean differences, demonstrating high variability in neutrophil counts among groups. Furthermore, the p-value for the ANOVA is <0.001 (p < 0.001), indicating a significant difference in neutrophil counts between the three groups. This implies that neutrophil numbers can vary greatly depending on the initial risk assessment using the HEART score, with higher counts indicating higher risk categories. The results demonstrate a clear association between neutrophil count and the HEART score risk category, with higher neutrophil counts observed in patients categorized as higher risk. Elevated neutrophil counts have been linked to increased inflammation and plaque destabilization in coronary arteries, contributing to the pathogenesis of ACS. Therefore, the significant increase in neutrophil counts in higher-risk categories suggests a heightened inflammatory response and a potentially greater risk of adverse cardiovascular events in these patients. These findings emphasize the importance of considering inflammatory markers, such as neutrophil counts, in risk stratification and management strategies for patients with suspected ACS.

**Table 4:** Comparison of neutrophil count and HEART score risk category by using one-way ANOVA Group 1: low-risk HEART score patients, Group 2: moderate-risk HEART score patients, Group 3: high-risk HEART score patients; 95% CI: confidence intervals (95%); HEART: history, ECG, age, risk factors, and troponin; ANOVA: analysis of variance Values are expressed in mean ± SD and the sample size is represented as 'N' One-way ANOVA test: *p < 0.05, **p < 0.001 (statistically significant), ns (not significant)

Parameter	Group 1 - Mean ± SD (95% CI), N = 123	Group 2 - Mean ± SD (95% CI), N = 94	Group 3 - Mean ± SD (95% CI), N = 71	F-value	p-value
Neutrophil count (%)	70.80 ± 9.33 (70.099 - 71.501)	77.59 ± 7.84 (76.915 - 78.265)	82.36 ± 6.96 (81.812 - 82.908)	46.350	<0.001**

Table 5 represents the mean and SD of lymphocyte counts for three groups categorized by the HEART score risk category. The mean lymphocyte count falls as the HEART score risk group increases, with Group 1 having the highest mean count of 22.05%, followed by Group 2 with a mean of 15.8%, and Group 3 with the lowest mean of 11.30%. These mean differences are substantiated by the estimated F-value of 60.264, which indicates high variability in lymphocyte counts between groups. The ANOVA shows a significant difference in lymphocyte counts among the three groups (p < 0.001). This implies that lymphocyte count may change greatly based on the initial risk assessment using the HEART score. The lower counts are associated with greater risk categories. The results demonstrate a clear association between lymphocyte count and the HEART score risk category, with lower lymphocyte counts observed in patients categorized as higher risk. The decrease in lymphocyte counts with increasing risk suggests a dysregulated immune response and potential immunosuppression in patients with more severe cardiovascular disease. Furthermore, lower lymphocyte counts may reflect underlying systemic inflammation and oxidative stress, both of which are implicated in the pathogenesis and progression of ACS. Lymphopenia has been identified as an independent predictor of mortality and adverse cardiac events in ACS patients, highlighting its prognostic significance.

**Table 5:** Comparison of lymphocyte count and HEART score risk category by using one-way ANOVA Group 1: low-risk HEART score patients, Group 2: moderate-risk HEART score patients, Group 3: high-risk HEART score patients; 95% CI: confidence intervals (95%); HEART: history, ECG, age, risk factors, and troponin; ANOVA: analysis of variance Values are expressed in mean ± SD and the sample size is represented as 'N' One-way ANOVA test: *p < 0.05, **p < 0.001 (statistically significant), ns (not significant)

Parameter	Group 1 - Mean ± SD (95% CI), N = 123	Group 2 - Mean ± SD (95% CI), N = 94	Group 3 - Mean ± SD (95% CI), N = 71	F-value	p-value
Lymphocyte count (%)	22.05 ± 8.00 (21.121 - 22.979)	15.8 ± 6.01 (14.969 - 16.631)	11.30 ± 5.20 (10.564 - 12.036)	60.264	<0.001**

Table 6 represents the mean and SD of platelet counts for three groups categorized by the HEART score risk category. Group 3 has the highest mean platelet count (285.94 x 10^9/µL), followed by Group 2 (260.06 x 10^9/µL) and Group 1 (248.35 x 10^9/µL). These mean differences are supported by the computed F-value of 5.211, indicating significant variability in platelet counts between groups. The ANOVA results show a significant difference in platelet counts among the three groups (p < 0.001). This implies that platelet counts may differ dramatically depending on the initial risk assessment using the HEART score, with larger numbers associated with higher risk categories. The results demonstrate a clear association between platelet count and the HEART score risk category, with higher platelet counts observed in patients categorized as higher risk. Platelets play a central role in hemostasis and thrombosis, and elevated platelet counts have long been recognized as a risk factor for thrombotic events in cardiovascular disease. The observed increase in platelet counts with increasing risk categories suggests a heightened thrombotic propensity and a potentially greater risk of acute thrombotic events in high-risk ACS patients.

**Table 6:** Comparison of platelet count and HEART score risk category by using one-way ANOVA Group 1: low-risk HEART score patients, Group 2: moderate-risk HEART score patients, Group 3: high-risk HEART score patients; 95% CI: confidence intervals (95%); HEART: history, ECG, age, risk factors, and troponin; ANOVA: analysis of variance Values are expressed in mean ± SD and the sample size is represented as 'N' One-way ANOVA test: *p < 0.05, **p < 0.001 (statistically significant), ns (not significant)

Parameter	Group 1 - Mean ± SD (95% CI), N = 123	Group 2 - Mean ± SD (95% CI), N = 94	Group 3 - Mean ± SD (95% CI), N = 71	F-value	p-value
Platelet count (10^9^/µl)	248.35 ± 58.14 (243.428 - 253.272)	260.06 ± 63.86 (254.670 - 265.450)	285.94 ± 73.63 (277.605 - 294.275)	5.211	0.006**

Table 7 represents NLR across three groups categorized by the HEART score risk category. The mean NLR rises dramatically as the HEART score risk category increases. Patients in Group 3 (high-risk) have the highest mean NLR of 9.20 ± 2.69, followed by Group 2 (6.04 ± 3.50) and Group 1 (3.86 ± 2.03). These mean differences are supported by the estimated F-value of 56.579, indicating significant variation in NLR between groups. The ANOVA results show a significant difference in NLR across the three groups (p < 0.001). This implies that NLR may vary greatly based on the initial risk assessment using the HEART score, with greater NLR values associated with higher risk categories. The results indicate a robust relationship between NLR and the HEART score risk category, with increased NLR reported in patients classified as higher risk. The robust relationship observed between increasing NLR and higher risk categories, as demonstrated in our study, underscores the potential utility of NLR as a prognostic marker in ACS. NLR reflects the balance between systemic inflammation (neutrophils) and immune surveillance (lymphocytes), and elevated NLR values have been associated with increased inflammatory response and poor outcomes in various cardiovascular conditions, including ACS. The observed increase in NLR with higher risk categories suggests a heightened inflammatory state and a potentially greater risk of adverse cardiovascular events in high-risk ACS patients. Moreover, NLR has emerged as a valuable prognostic marker in ACS, with higher NLR values associated with increased mortality, recurrent myocardial infarction, and MACEs. NLR provides additive prognostic information beyond traditional risk factors and biochemical markers, aiding in risk stratification and guiding clinical decision-making in ACS patients.

**Table 7:** Comparison of neutrophil lymphocyte ratio (NLR) and HEART score risk category by using one-way ANOVA Group 1: low-risk HEART score patients, Group 2: moderate-risk HEART score patients, Group 3: high-risk HEART score patients; 95% CI: confidence intervals (95%); HEART: history, ECG, age, risk factors, and troponin; ANOVA: analysis of variance Values are expressed in mean ± SD and the sample size is represented as 'N' One-way ANOVA test: *p < 0.05, **p < 0.001 (statistically significant), ns (not significant)

Parameter	Group 1 - Mean ± SD (95% CI), N = 123	Group 2 - Mean ± SD (95% CI), N = 94	Group 3 - Mean ± SD (95% CI), N = 71	F-value	p-value
Neutrophil lymphocyte ratio (NLR)	3.86 ± 2.03 (3.630 - 4.090)	6.04 ± 3.50 (5.320 - 6.760)	9.20 ± 2.69 (8.870 - 9.570)	56.579	<0.001**

Table 8 represents PLR across three groups categorized by the HEART score risk category. The mean PLR rises dramatically as the HEART score risk category increases. Group 3 (high-risk patients) has the greatest mean PLR (268.94 ± 100.76), followed by Group 2 (159.76 ± 33.78) and Group 1 (103.71 ± 37.12). These mean differences are supported by the estimated F-value of 177.95, demonstrating significant variation in PLR between groups. The ANOVA results show a statistically significant difference in PLR across the three groups (p < 0.001). This implies that PLR may vary greatly based on the initial risk assessment using the HEART score, with greater PLR values associated with higher risk categories. The results indicate a robust relationship between PLR and the HEART score risk category, with higher PLR reported in patients classified as higher risk. The strong association observed between increasing PLR and higher risk categories, as demonstrated in our study, underscores the potential of PLR as a valuable prognostic tool in ACS. PLR serves as a composite marker of platelet activity and systemic inflammation, reflecting the interplay between thrombosis and inflammation in the pathogenesis of ACS. The observed increase in PLR with higher risk categories suggests a prothrombotic and proinflammatory milieu in high-risk ACS patients, predisposing them to adverse cardiovascular outcomes. PLR has emerged as a promising prognostic tool in ACS, with higher PLR values associated with increased mortality, recurrent myocardial infarction, and MACEs. PLR provides incremental prognostic value beyond traditional risk factors, biochemical markers, and imaging modalities, aiding in risk stratification and guiding clinical decision-making in ACS patients.

**Table 8:** Comparison of platelet lymphocyte ratio (PLR) and HEART score risk category by using one-way ANOVA Group 1: low-risk HEART score patients, Group 2: moderate-risk HEART score patients, Group 3: high-risk HEART score patients; 95% CI: confidence intervals (95%); HEART: history, ECG, age, risk factors, and troponin; ANOVA: analysis of variance Values are expressed in mean ± SD and the sample size is represented as 'N' One-way ANOVA test: *p < 0.05, **p < 0.001 (statistically significant), ns (not significant)

Parameter	Group 1 - Mean ± SD (95% CI), N = 123	Group 2 - Mean ± SD (95% CI), N = 94	Group 3 - Mean ± SD (95% CI), N = 71	F-value	p-value
Platelet lymphocyte ratio (PLR)	103.71 ± 37.12 (95.748 - 111.772)	159.76 ± 33.78 (151.726 - 167.794)	268.94 ± 100.76 (244.613 - 293.267)	177.95	<0.001**

## Discussion

Non-ST elevation acute coronary syndrome (NSTE-ACS) poses significant challenges in the emergency department (ED) due to the need for prompt risk stratification and management decisions. The HEART score has emerged as a valuable tool for risk assessment in patients presenting with chest discomfort, guiding clinicians in identifying individuals at high risk who require urgent intervention. In our study, we investigated the relationship between PLR and NLR in patients with NSTE-ACS and their HEART score, aiming to provide insights into their prognostic value in this clinical setting. Our study revealed a favorable relationship between mean PLR and NLR and HEART score in patients with NSTE-ACS, with significantly higher values observed in the high-risk HEART score group. These findings align with previous research highlighting the prognostic significance of PLR and NLR in cardiovascular diseases.

In the ED, risk stratification needs to be given clinical attention. In higher-risk cases, clinical guidelines advise an early invasive approach in NSTE-ACS [10]. In several nations, the HEART score is a proven quick risk stratification measure for individuals experiencing chest discomfort. This helps doctors identify high-risk patients who require immediate attention [11]. In our study, we discovered a favorable relationship between the mean PLR and NLR of patients admitted with NSTE-ACS and their HEART score. The high-risk HEART score group showed significantly increased PLR and NLR.

Because leukocytes affect the instability of atherosclerotic plaques, they are important in the pathophysiology of ACS. Leukocytes enter endothelial cells during the first stage and activate upon reaching the tunica intima. They cause microvasculature to form there, which increases the likelihood of plaque rupture [12]. In a study by Sabatine et al., individuals with ACS (UA, NSTEMI) who had an elevated WBC count were found to have a relevant risk factor for death over the first 30 days and six months after myocardial infarction [13]. Polymorphonuclear (PMN) cells have been seen in coronary thrombi in several myocardial infarction patients following primary percutaneous coronary intervention. At the site of the causing lesion, PMN releases neutrophil extracellular traps (NETs). Leukocytes can be ensnared by NETs, which are strongly prothrombotic and proinflammatory fibers that spread thrombosis. It was shown that NETs had a positive correlation with infarct size and a negative correlation with ST-segment resolution [14]. In contrast, components of the adaptive immune system, particularly B2 and T helper cells can suppress and reduce inflammation. Reduced lymphocyte numbers were linked to worse clinical outcomes and the advancement of atherosclerosis in patients with heart failure and ACS [15]. The high-risk HEART score group in our study had mean PLR and NLR that were significantly greater than those of the other two groups, and both had a positive correlation with the HEART score.

In a four-year follow-up study, Azab et al. examined the mortality of NSTEMI patients after four years. They discovered that PLR was an independent predictor of mortality after four years, indicating that high PLR is a predictor of long-term rather than just a marker of an acute medical condition [16]. Platelets contribute to inflammation and play evident roles in thrombosis. Activated platelets aid neutrophil adhesion to the subendothelial matrix during stress. According to Chirkov et al., patients with stable angina pectoris and ACS have higher platelet aggregability and nitric oxide resistance than those without coronary heart disease (CHD) [17]. Our study aligns with the findings of Azab et al. by corroborating the significance of PLR as a predictor of long-term mortality in NSTEMI patients. Both studies highlight the importance of PLR beyond its acute implications, emphasizing its utility as a prognostic indicator for future adverse outcomes. Similarly, our study resonates with the observations of Chirkov et al. regarding the role of platelets in inflammation and thrombosis. By demonstrating elevated PLR and NLR in patients with high-risk HEART scores, our study reinforces the notion that these markers reflect underlying inflammatory processes and thrombotic tendencies. Our findings, thus, lead us to conclude that PLR and NLR are simple, low-cost methods for identifying ED patients with high-risk HEART scores.

Limitations of the study

Firstly, the study's single-center design might restrict the generalizability of the findings, as patient demographics, management protocols, and outcomes could differ in other healthcare settings. Furthermore, the relatively small sample size may have limited our ability to detect minor yet clinically significant associations within the data. Moreover, selection bias might have influenced the results due to the specific inclusion and exclusion criteria employed. The cross-sectional nature of the study limits the ability to establish causality or temporal relationships between PLR/NLR and high-risk HEART scores, necessitating further longitudinal investigations. Furthermore, while efforts were made to control for potential confounders, such as age, comorbidities, and medications, residual confounding may still exist. Statistical considerations, such as multiple comparisons and adjustment methods, should be carefully evaluated to ensure the robustness of the results. Addressing these limitations in future research endeavors is paramount. Collaborative efforts involving multiple centers would enhance the diversity and representativeness of study populations, thereby increasing the external validity of findings. Larger sample sizes would improve statistical power and facilitate the detection of smaller but clinically relevant associations. Prospective study designs with longer follow-up periods would enable the examination of temporal relationships and the prediction of future clinical events. Comprehensive adjustment for confounders and careful consideration of statistical methods, such as controlling for multiple comparisons and employing appropriate adjustment techniques, would strengthen the validity and reliability of study results. By addressing these limitations, future research can provide more robust evidence on the prognostic significance of PLR and NLR in patients with NSTEMI and UA, ultimately informing clinical practice and patient care.

## Conclusions

We present compelling evidence indicating a robust positive correlation between the HEART score, PLR, and NLR in patients with NSTEMI and UA. Our study underscores the critical importance of predictive markers in expediting treatment interventions, particularly given the persistently high mortality rate associated with NSTEMI. Notably, PLR and NLR emerge as independent predictors of long-term adverse clinical outcomes, shedding light on their utility in risk stratification. By emphasizing the predictive significance of PLR and NLR, our research contributes to a deeper understanding of the molecular mechanisms underlying inflammation and leukocyte activity in ACS, thereby offering valuable insights for clinical practice.

Furthermore, our findings hold particular relevance in resource-limited settings, such as those often encountered in developing countries like India. PLR and NLR emerge as simple, cost-effective, and accurate early predictors of high-risk HEART score patients, thus filling a crucial gap in risk assessment strategies. This highlights their potential to significantly impact clinical decision-making and improve patient outcomes, especially in settings where access to advanced diagnostic tools may be limited. Moving forward, our study encourages further research to explore the broader applicability of these biomarkers and their integration into clinical practice guidelines. By harnessing the predictive power of PLR and NLR, healthcare providers can make informed decisions tailored to individual patient needs, ultimately advancing the management of ACS and enhancing patient care worldwide.
